# Entropy effects in the collective dynamic behavior of alkyl monolayers tethered to Si(111)

**DOI:** 10.3762/bjnano.6.60

**Published:** 2015-02-26

**Authors:** Christian Godet

**Affiliations:** 1 Institut de Physique de Rennes, UMR 6251 CNRS - Université de Rennes 1, 35042 Rennes Cedex, France

**Keywords:** admittance spectroscopy, dipolar relaxation, entropy, gauche defect, organic monolayer

## Abstract

Dynamic properties of *n*-alkyl monolayers covalently bonded to Si(111) were studied by broadband admittance spectroscopy as a function of the temperature and the applied voltage using rectifying Hg/C_12_H_25_/n-type Si junctions. Partial substitution of methyl end groups by polar (carboxylic acid) moieties was used to enhance the chain end relaxation response. Two thermally activated dissipation mechanisms (B1 and B2, with *f*_B1_ < *f*_B2_) are evidenced for all reverse bias values. The strong decrease of both relaxation frequencies with increasing reverse dc bias reveals increasing motional constraints, attributed to electrostatic pressure applied to the densely-packed nanometer-thick monolayer. Spectral decomposition of the frequency response shows a power-law dependence of their activation energies on |*V*_DC_|. A large reverse bias reversibly increases the B2 response attributed to the distribution of gauche defects, in contrast with the constant strength of the acid dipole loss (B1). A trans–gauche isomerization energy of 50 meV is derived from the temperature dependence of the B2 dipolar strength. For both dissipation mechanisms, the observed linear correlation between activation energy and logarithm of pre-exponential factor is consistent with a multi-excitation entropy model, in which the molecular reorientation path is strongly coupled with a large number of low energy excitations (here the *n*-alkyl bending vibrational mode) collected from the thermal bath. This collective dynamic behavior of alkyl chains tethered to Si is also confirmed by the asymmetric relaxation peak shape related to many-body interactions in complex systems.

## Introduction

Self-assembled monolayers (SAM) and organic molecular layers (OML) have attracted great interest over the past two decades because surface functionalization offers great flexibility for a molecular-level control of surface chemistry, surface energy, biocompatibility, friction, corrosion, liquid chromatography, interfacial interactions and electronic transport [1–6]. More recent studies have been focused on the functionalization of nanostructures. However, in spite of a large number of experimental and simulation studies, it is not yet clear how the layer structure affects the measured properties of such functionalized surfaces. In particular, a number of structural and conformational models have been proposed to describe mechanical [7–24] and electron transport [7,25–32] properties.

In order to understand the behavior of nanometer-thick 2D assemblies of molecules tethered to metallic or semiconductor surfaces, the concepts developed for bulk organic solids should be revisited by also considering molecular coverage, lateral (in-plane) inhomogeneity and transverse gradient of disorder [3].

As far as disorder is concerned, in model systems made of linear alkyl chains tethered in a densely packed array, experiments and simulations indicate that a quasi-perfect order can be obtained (at least locally) at low temperature, in the most stable all-trans conformation of the (CH_2_)*_n_* molecular backbone [17,22]. However, increasing temperature has a strong influence on the tethered polymethylene OML structure because new chain configurations can be reached from this free energy minimum by a trans–gauche isomerization mechanism (rotation around the C–C axis). Hence, although some topological order is imposed by the head group binding at particular sites of a crystalline substrate, orientational order induced by lateral chain interactions (e.g., van der Waals, electrostatic and dipolar forces) is progressively lost for chain segments located away from the head towards the molecular tail. The strain-induced formation of gauche defects, initiating at outer bonds (end-gauche) and proceeding inward (kinks and gauche–gauche conformers) [3,17,22] results into a disorder gradient.

Another important issue related to energy dissipation mechanisms is the behavior of tethered OML under compressive and shear forces, as found in nano-tribology experiments, where external forces can cause conformational changes. Again, a disorder gradient results from the formation of gauche defects which can be reverted when the atomic force microscope (AFM) tip is moved away (laterally or vertically) [7,9–16,18,21,23–24].

In the field of molecular electronics, many studies were performed by using junctions made of alkyl OML tethered to oxide-free silicon surfaces through chemically stable non-polar Si–C bonds [4,33–35]. These robust densely packed insulating molecular layers play the role of nanometer-thick tunnel barriers [25,27,31–32,36–39]. Although conformational changes are intrinsic to soft matter, the consequences of temperature-induced [25–27,31] and pressure-induced [7,9] conformational changes on electron transport properties have rarely been explicitly described.

In this context, this admittance spectroscopy study emphasizes a collective dynamic behavior of linear saturated (*n*-alkyl) chains tethered to Si(111). Dynamic properties are very sensitive to structure and conformation of the OML. In contrast with alternative dynamic probes, such as nuclear magnetic resonance, which are limited by a poor signal-to-noise ratio and require functionalization of 3D nanoparticles or porous solids [3], admittance spectroscopy is sensitive to 0.3 picomoles of carboxylic acid dipoles [40] and measurements can be performed in a well-defined metal/OML/semiconductor planar configuration, which is relevant for molecular electronics devices. Admittance spectroscopy provides insights in the modulation of localized charge density and dipole reorientation in a system submitted to a time-dependent electric field. Dissipation (energy loss) mechanisms can be described by using equivalent representations of the complex admittance, including the dielectric permittivity ε* and electrical modulus M*. Dipole reorientation requires an activation of the system with energy barriers related either to local or more collective reorientation mechanisms.

Previous admittance spectroscopy studies of metal/*n*-alkyl/Si junctions have shown changes in peak shape and frequency of the molecular relaxation signature with increasing forward bias, which were attributed to an enhanced rigidity of OML [29–30]. Recently, the temperature dependence of the molecular relaxation frequency (at low reverse bias) has revealed the sensitivity of its activation energy to end-group functionalization, namely increased motional constraints with carboxylic acid substitution to methyl groups [40].

**Figure 1:**
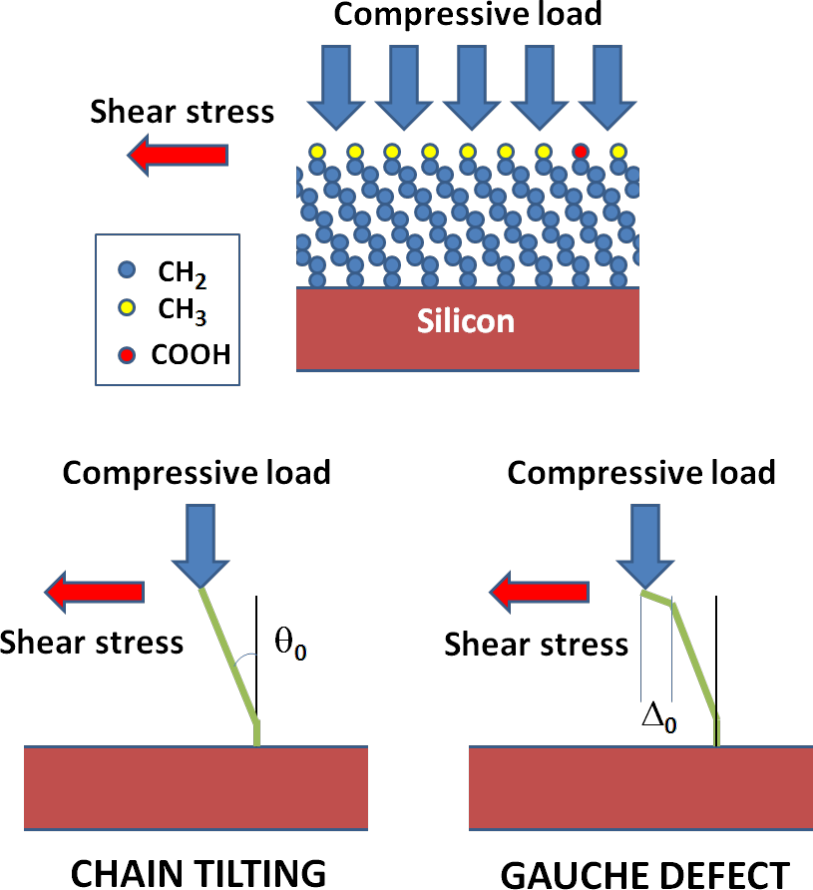
Top: the electrostatic pressure resulting from a dc voltage drop on the insulating molecular monolayer is described by a normal compressive load. Bottom: the mechanical response to the induced shear stress may lead to global chain axis tilting (left panel) or formation of gauche defects preferentially localized at chain ends (right panel).

This extension of our previous work [40] to large reverse dc bias applied to Hg/C_12_H_25_/Si tunnel junctions, resulting in a change in the distribution of topological defects, reveals a collective behavior of linear saturated (*n*-alkyl) chains tethered to the Si(111) surface. The effects of both temperature and applied dc bias on the dynamics of a monolayer of *n*-alkyl chains tethered to Si(111) are studied by broadband admittance spectroscopy (0.1 Hz to 10 MHz) using a partial substitution of methyl (CH_3_) end groups (0.1 Debye) by carboxylic acid (COOH) dipolar moieties (1.74 Debye) in order to enhance the chain end relaxation response. Here, a mixed alkyl/acid-functionalized monolayer with 5% acid molar fraction (in the liquid phase) was chosen to avoid acid–acid dipole interactions at the OML surface (Figure 1). Using low-doped n-type Si (1–10 Ω·cm) provides strong rectification [27,32,38–40] with a very low dc current in the reverse bias regime at low temperatures (130–300 K). In addition, low doping is interesting for providing a low depletion capacitance, which increases the high frequency cutoff (typically 1 MHz) arising from the series resistance *R*_S_ (due to bulk Si and rear contact resistance) [40–41].

In the experimental admittance *Y*(*V*,*T*,ω) characteristics, two bias-dependent relaxation peaks (B1 and B2, with *f*_B1_ < *f*_B2_) are observed in addition to a bias-independent peak A near 10^3^ Hz, previously attributed to adventitious water condensation [32,40]. With increasing reverse bias, the slowing down of both relaxation frequencies *f*_B1_ and *f*_B2_ reveals increasing motional constraints, which are attributed to electrostatic pressure effects. The magnitude of this electrostatic pressure remains well below the applied tip pressure used in AFM experiments (0.03–60 GPa) [21,24]. Since the dipolar relaxation peaks, B1 and B2, overlap with peak A, a spectral decomposition is mandatory to obtain the intensities and frequencies of dipolar relaxation peaks, along with the asymmetric peak shapes in the context of Dissado–Hill (DH)/Jonscher theories for many-body interactions [42–46]. Arrhenius plots of the relaxation frequencies show that the apparent activation energies and pre-exponential factors strongly increase with applied dc voltage, |*V*_DC_|.

The dynamics of the tethered OML will be discussed in terms of a collective behaviour, with three complementary approaches: a) The DH model addresses spatial correlations between “embedded dipoles” in order to describe the asymmetric relaxation peak shapes [45–46]. b) The multi-excitation entropy model proposed by Yelon, Movaghar and Crandall (YMC) [47–48] considers a thermodynamic description of the dipole reorientation path strongly coupled with elementary excitations collected from a thermal bath. c) The applied bias dependence of activation energies is tentatively related to compression and shear stresses expected for an OML considered as a continuous medium submitted to a compressive force (Figure 1) [49]. Finally, on the basis of the bias dependence of the relaxation peak intensities, relaxation mechanisms B1 and B2 will be, respectively, attributed to acid end-group dipoles and to gauche defect configurations.

## Results

As reported previously [32], several techniques were used to obtain complementary information on the conformal coverage (STM, AFM), OML thickness (spectroscopic ellipsometry, SE), molecular packing density and possible interface oxidation of the Si substrate (X-ray photoelectron spectroscopy). The surface density of acid groups (0.4 × 10^14^ cm^−2^) and the total organic layer (acid + alkyl) coverage (2.6 × 10^14^ cm^−2^) were obtained by XPS using, respectively, the C 1s (COOH) signal and the total C 1s peak area. The thickness (*d*_SE_ = 1.06 ± 0.1 nm) obtained from SE data indicates a rather large average tilt angle (40°) of the chain axis with respect to the normal direction.

Dipolar relaxation is investigated in a wide frequency range (from 1 × 10^−1^ Hz to 1 × 10^7^ Hz). To observe dipolar relaxation mechanisms, low temperature and reverse bias were imposed to obtain low dc conductance and a relaxation frequency of the space-charge layer below 1 Hz [32]. The complex admittance, Y*(*V*_DC_,*T*,ω) = *G*_m_ + jω *C*_m_, can be analyzed by using either the capacitance C* = (Y*/jω) or the electrical modulus M* = (ε*)^−1^ = jω *C*_0_/Y* (here *C*_0_ = C*/ε* is arbitrarily set to 100 pF). The characteristic frequencies of the loss peaks in imaginary modulus, *M*″(ω), or in imaginary permittivity, ε″(ω), data correspond to a delay between the electric field and local charge modulation or dipole rotation [32,39–40,50].

In the following, the low dc bias situation is briefly recalled, before considering the effect of increasing the reverse bias, |*V*_DC_|, and the spectral analysis method.

### Dipolar relaxation at low applied bias

**Figure 2:**
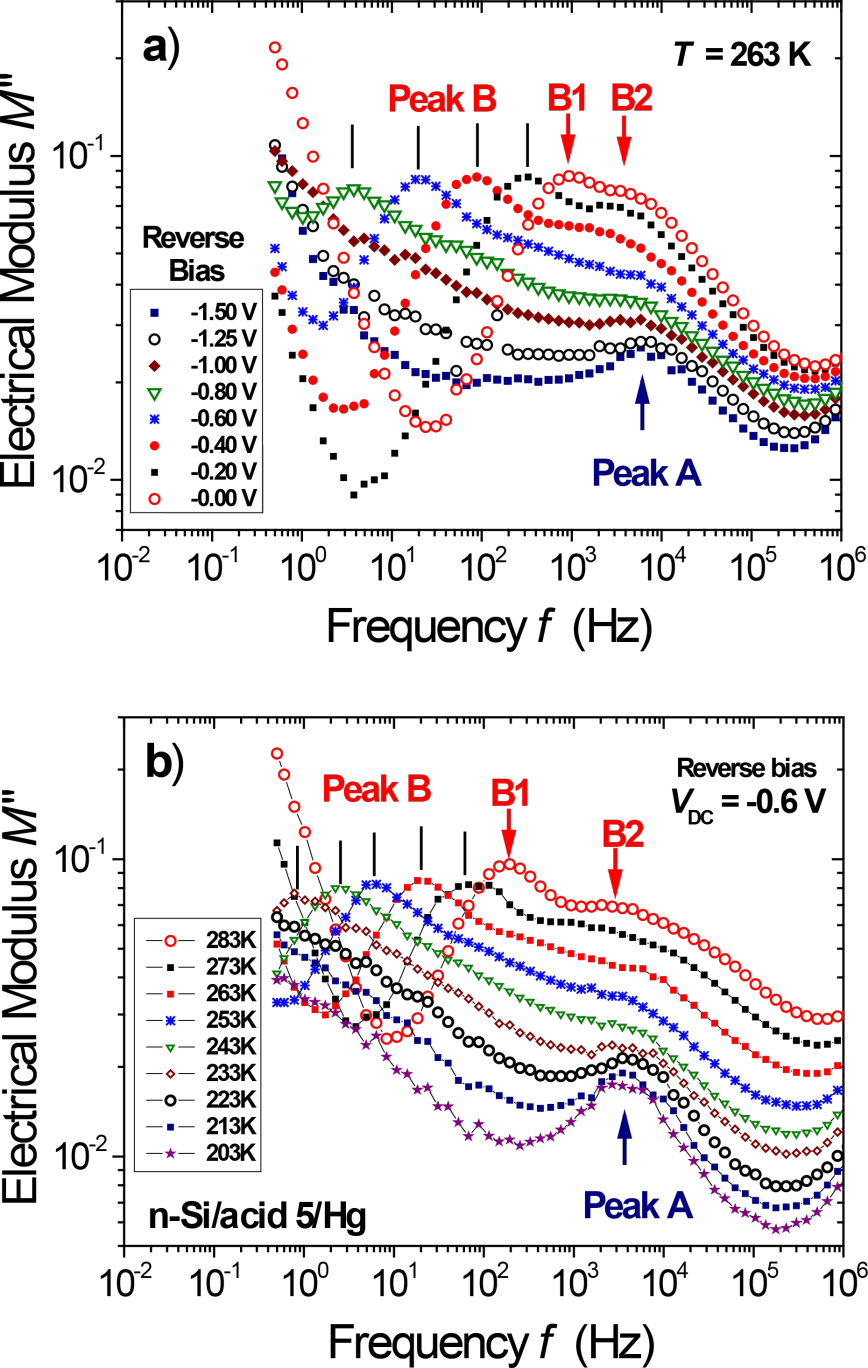
Imaginary electrical modulus *M*″(ω) of the Hg/acid 5**/**n-type Si junction: a) reverse-bias dependence at *T* = 263 K; b) temperature dependence (203–283 K) under strong reverse bias (*V*_DC_ = −0.6 V). The admittance data show (i) a bias-independent relaxation peak A (*f*_A_ ≈ 5 kHz) with a weak *T* dependence, and (ii) two bias-dependent peaks (B1 and B2) at lower frequency with strong bias and temperature dependence.

Two classes of relaxation mechanisms, A and B, have been identified with, respectively, a weak (*f*_A_) and a strong (*f*_B_) temperature dependence of the relaxation frequencies [32]. At low temperatures (*T* < 150 K), only mechanism A is observed at intermediate frequencies (*f*_A_ ≈ 4 × 10^3^ Hz in Figure 2b). The characteristic frequency *f*_A_ is basically bias-independent and has very small values for activation energy (*E*_A_ = 29.6 ± 1 meV) and pre-exponential factor (*f*_A_^0^ ≈ (7 ± 2) × 10^3^ Hz). Its small modulus intensity, *M*″_MAX_ ≈ 0.02, and dipolar relaxation strength, Δε ≈ 0.09, decrease weakly as a function of the increasing temperature (Figure 6b in [32]). The asymmetric *M*″(ω) peak shape is defined by an apparent pre-peak slope *m*_DH_(A) ≈ 0.86 ± 0.05 and a post-peak slope (1 − *n*_DH_(A)) ≈ 0.61 ± 0.05.

With increasing temperature mechanism B also appears. As a matter of fact, two relaxation peaks B1 and B2 (*f*_B1_ < *f*_B2_) are clearly observed in junctions with diluted carboxyl end group dipoles (Si/acid 5), a larger *M*″_MAX_ intensity being found for the lower-frequency B1 peak (Figure 2). For the stronger peak B1, the relaxation frequency *f*_MAX_ taken at the *M*″_MAX_ peak maximum exhibits an activated behavior *f*_MAX_ = *f_B_*^0^ × exp(−*E*_B_/*kT*) with high values for the activation energy, *E*_B_ ≈ 0.40 eV, and the pre-exponential factor (*f_B_*^0^ ≈ 10^10^ Hz) [32]. As shown below, the spectral decomposition method (*E*_B1_ = 0.37 eV) confirms this result; at low bias, a small but significant difference in activation energy values is also found between mechanisms B1 (*E*_B1_ = 0.37 eV) and B2 (*E*_B2_ = 0.32 eV). In the following, bias-dependent relaxation data will be helpful to elucidate their identification.

### Bias dependence of dipolar relaxation

With increasing reverse bias, a strong slowing down of both relaxation mechanisms at frequencies *f*_B1_ and *f*_B2_ is observed. Figure 2a illustrates this behavior at *T* = 263 K, along with the constant strength of the B1 relaxation mechanism. In the reverse-bias regime, both B1 and B2 peak frequencies show a stronger temperature dependence, as illustrated in Figure 2b, at *V*_DC_ = −0.6 V. The ratio between the respective frequencies of peaks B1 and B2 being less than 20 for all investigated temperatures, they strongly overlap. Note that peak A is also present in the available frequency window, although it clearly appears only at very low temperature and strong reverse bias.

#### Spectral decomposition

The analysis of the relaxation data requires some fitting of admittance spectra with a large number of unknown parameters (characteristic frequencies, peak intensity *M*″_MAX_ or dipolar relaxation strength Δε, peak shape exponents). Hence, measurements with wide frequency windows at low temperatures are crucial experimental conditions. To describe the relaxation peak shapes, we assume that all three relaxation mechanisms follow the same type of many-body interactions (although with different parameters) consistent with a collective response of the densely-packed assembly of tethered organic chains.

To account for the typically observed deviations from the ideal Debye relaxation, Dissado–Hill and Jonscher have elaborated the theoretical description of many-body interactions in complex systems on the basis of an ideal structure that interlinks the dipoles and can be represented by perturbations on different length scales [45–46]. The relaxation rate β_iN_ of an individual dipole is defined by its interactions within a cluster of size *N*_i_ formed by its surrounding inactive neighbors. The occurrence of correlated-cluster regions with sizes *M*_j_ depending on the strength of dipolar screening [42,44] takes place at the mesoscopic level. The macroscopic average over all cooperative mesoscopic regions provides the universal relaxation, given by the dielectric susceptibility [29–30,45–46,51]:

[1]
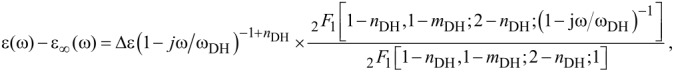


where the Gauss hypergeometric function _2_*F*_1_[ , ; ; ] is defined by 0 ≤ *m* ≤ 1 and 0 ≤ *n* ≤ 1, ω is the frequency and ω_DH_ is the peak frequency. Asymptotic limits, ω*^m^* for ω << ω_DH_ and ω*^n^*^−1^ for ω >> ω_DH_, are fully consistent with Jonscher [42–44] and Havriliak–Negami [52–53] expressions.

Fitting the four parameters (Δε, ω_DH_, *m*_DH_, *n*_DH_) for each relaxation mechanism is performed using the complex permittivity (Equation 1) by minimizing the error function EF(Δε, ω_DH_, *m*_DH_, *n*_DH_) given by the sum over the fitting range of [Ln (*M*″_EXP_/*M*″_CALC_)]^2^. In the following, we address the dependence of peaks B1 and B2 on temperature and bias, including their dipolar strength Δε = ε_0_ − ε_∞_, along with the shapes resulting from their collective response.

#### Admittance data analysis

**Fitting procedure:** Decomposition of admittance spectra into three asymmetric relaxation peaks (A, B1 and B2) was performed by adjusting the Dissado–Hill parameters (Equation 1), excluding data in the low-frequency region where the dc current is strong and data in the high frequency regime (*f* > 0.1 MHz) where series resistance effects are significant. Because each relaxation peak is defined by four characteristic parameters (frequency, dipolar relaxation strength and two characteristic slope exponents) an unambiguous decomposition is not straightforward.

Since the activation energy and the pre-exponential factor of the relaxation frequency *f*_A_(*T*) are readily obtained from low-temperature data, according to *f*_A_ (Hz) = 1.5 × 10^4^ exp(−0.0296/*kT*), the frequency of peak A can be extrapolated to higher *T* and fixed in the further parameter adjustment. Note that any error (e.g., underestimation) made in the fixed *f*_A_ value influences (decreases) the fitted *n*_B2_ value. Some parameter adjustment was allowed for the slopes (*m*_A_ = 0.9 ± 0.1, *n*_A_ = 0.65 ± 0.1) and the relaxation strength, Δε = 0.15 ± 0.05 of peak A.

**Figure 3:**
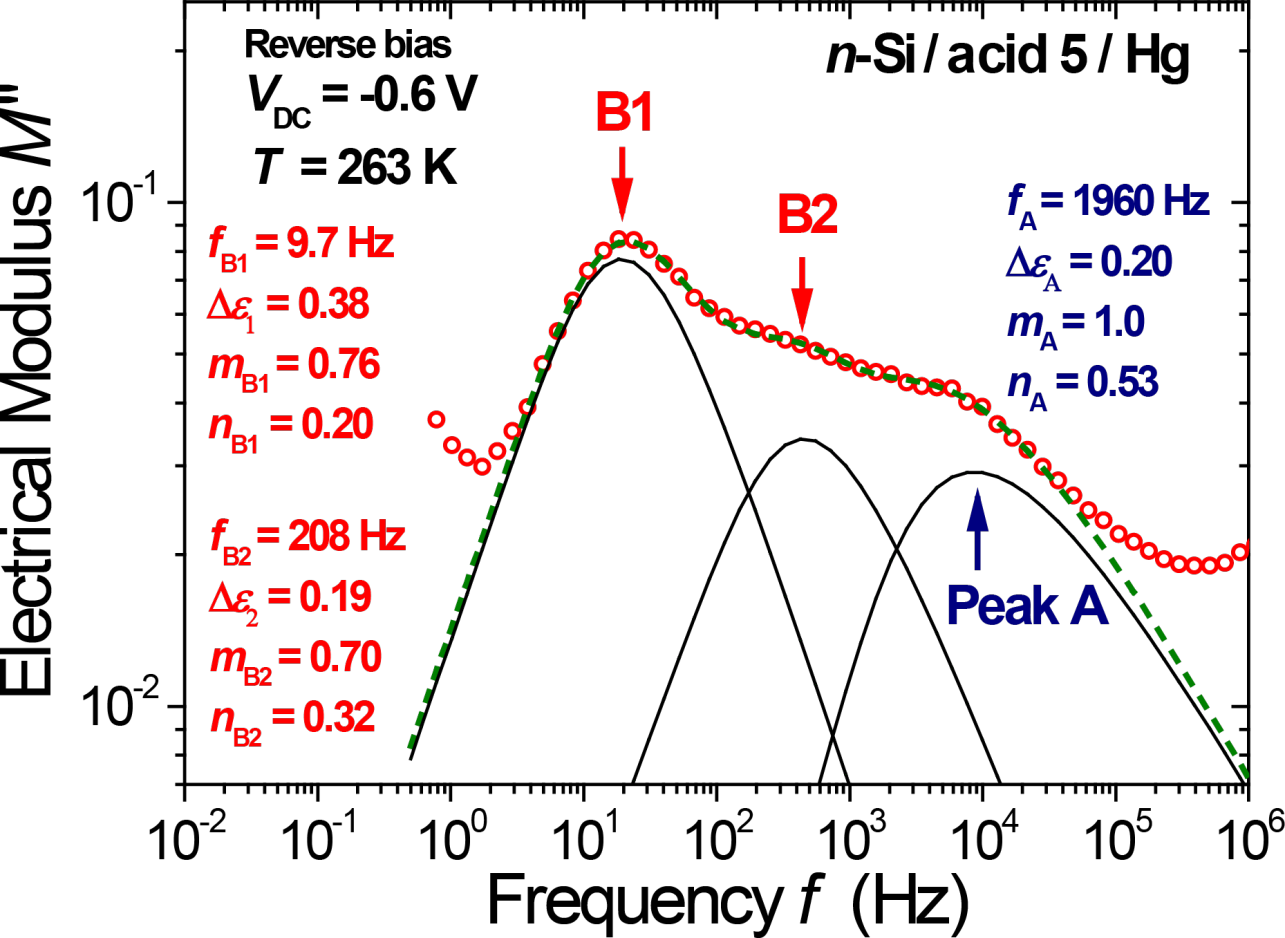
Decomposition of the electrical modulus *M*″(ω) obtained at *T* = 263 K and *V*_DC_ = −0.6 V into three dipolar relaxation peaks using the peak shape given by the Dissado–Hill theory (Equation 1). The dashed line is the peak sum fitted to the data (open circles).

In a first step of spectral decomposition, the slopes (*m*_DH_ and 1−*n*_DH_) were fixed in order to fit the two peak frequencies (*f*_B1_ and *f*_B2_) and the three peak strengths (A, B1 and B2). It was ensured that the change in the other (fitted) parameters remained small when the fixed *m*_B2_ value is changed by ±0.1 (typically at 0.7 or at 0.8). This constraint on *m*_B2_ is helpful to decrease some fluctuations in the fitted peak strength Δε as a function of the temperature. A representative example of data fitting is given in Figure 3 (with fixed values for *m*_A_ = 1 and *n*_A_ = 0.53) showing that an accurate decomposition into three peaks is obtained even for experimental spectra with substantial peak overlap; in this particular example, peaks B1 and B2 are separated by one decade (e.g., at *V*_DC_ = −0.6 V, the peak frequency ratio *f*_B2_/*f*_B1_= 20 ± 5).

In a second fitting step, the slopes were considered as additional free parameters to refine the spectral decomposition. The latter conditions improve the error function by a factor of about two. Note that the improvement of this error function value is limited by a few noisy data near 50 and 100 Hz.

**Peak shape:** The shapes of peaks B1 and B2 in log(*M*″) vs log(ω) plots are rather independent of the measurement temperature. However, pre-peak slopes must be compared in the higher *T* range, where *m*_B1_ is obtained with a good accuracy, while post-peak slopes must be compared in the lower *T* range, where *n*_B2_ is obtained with a good accuracy. In contrast, peak overlap provides larger error bars on the fitted *n*_B1_ and *m*_B2_ parameters.

The pre-peak slopes *m*_B1_ and *m*_B2_ are quite similar, and the small difference, *m*_B2_ ≈ 0.70 ≤ *m*_B1_ ≈ 0.78 ± 0.05 observed over many *T* and *V*_DC_ values, remains within the experimental and the fitting error. No systematic change of *m*_B1_ and *m*_B2_ with applied bias is detected. In the Dissado–Hill model, high values of pre-peak slopes indicate a large degree of disorder at the inter-cluster scale, i.e., at longer relaxation times.

In contrast, a larger difference appears in the fitted values of the post-peak slopes (*n*_B1_ ≈ 0.15 ± 0.1 << *n*_B2_ ≈ 0.6 ± 0.2). Although large errors bars are obtained for the fitted *n*_B2_ values, due to some frequency overlap with peak A, the difference is quite significant. In the Dissado–Hill model, a high value of the post-peak slope |*n*_B1_ − 1| for mechanism B1 indicates a large degree of disorder at the intra-cluster scale (i.e., at shorter relaxation times), approaching the situation *n* ≈ 0 where dipoles relax independently (leading to the Debye classical model, in contrast with *n* ≈ 1, where reorientations are fully correlated).

In summary, over the whole investigated temperature range, while little difference in long-range order (pre-peak slopes *m*_B1_ ≈ *m*_B2_) is observed between mechanisms B1 and B2, short-range order (post-peak slopes *n*_B1_ < *n*_B2_) is more developed for mechanism B2.

**Figure 4:**
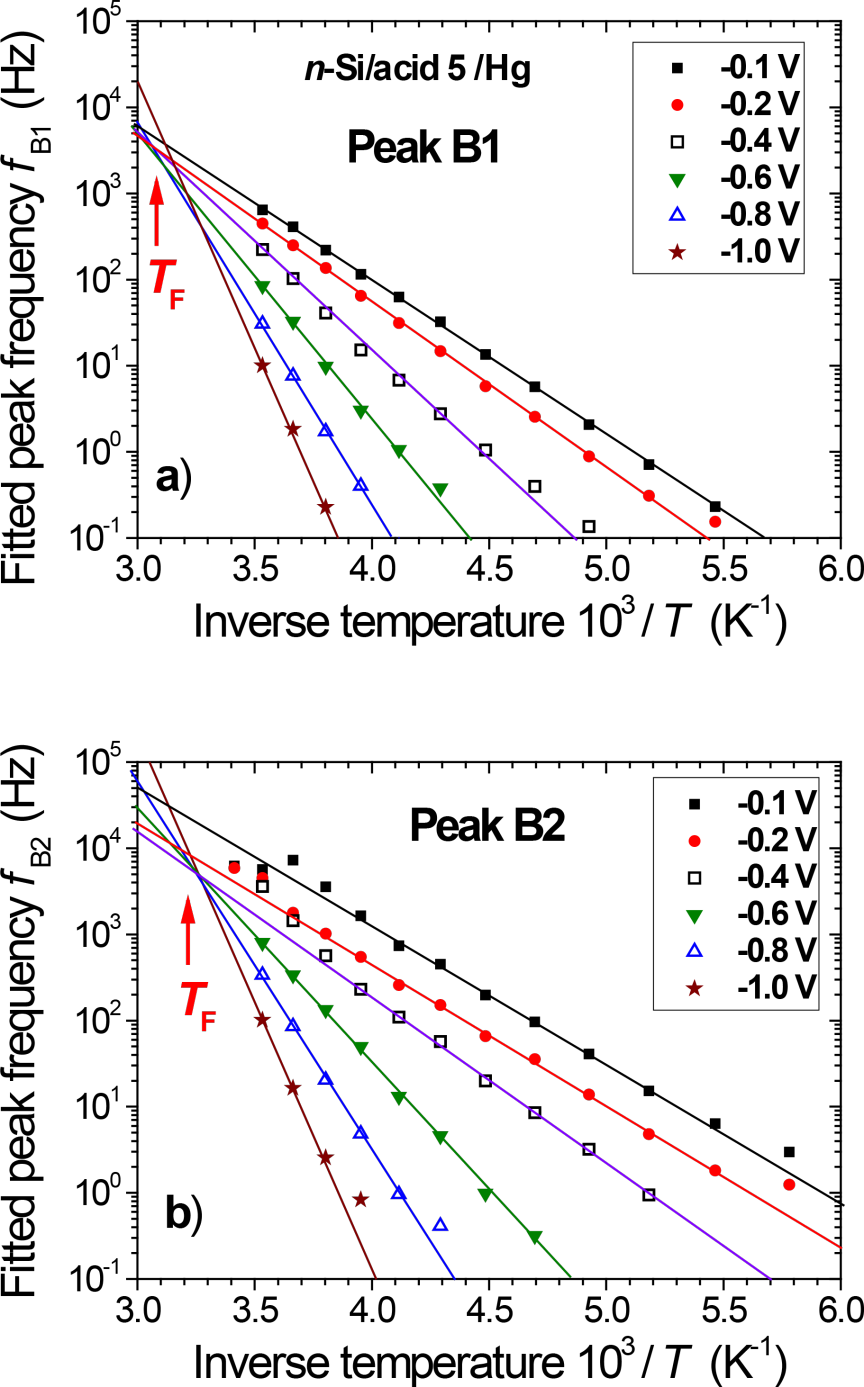
Temperature dependence of the dipolar relaxation frequencies *f*_B1_ (a) and *f*_B2_ (b) obtained for the Hg/acid 5**/**n-type Si junction at different reverse bias values (*V*_DC_ range from −0.1 V to −1.0 V). The lines are Arrhenius fits to the data, showing the existence of a "focal point" near *T*_F_ ≈ 320 K.

**Activation energy:** The activation energy of the relaxation peak frequency is interpreted in terms of motional constraints for dipole reorientation. For each *V*_DC_ value, the relaxation frequencies (*f*_B1_, *f*_B2_) reported in Figure 4 exhibit an Arrhenius dependence on the temperature, *f*_B_ = *f*_B_^0^ × exp(−*E*_B_/*kT*) over three decades. An important observation is the evidence of a so-called “focal point” at which the Arrhenius lines (extrapolated to high *T*) tend to converge; within the experimental error. A similar focal (or isokinetic) temperature *T*_F_ = 320 ± 10 K is found for both mechanisms B1 and B2 (Figure 4). The consequences of this result on the pre-exponential factor behavior are discussed below.

**Figure 5:**
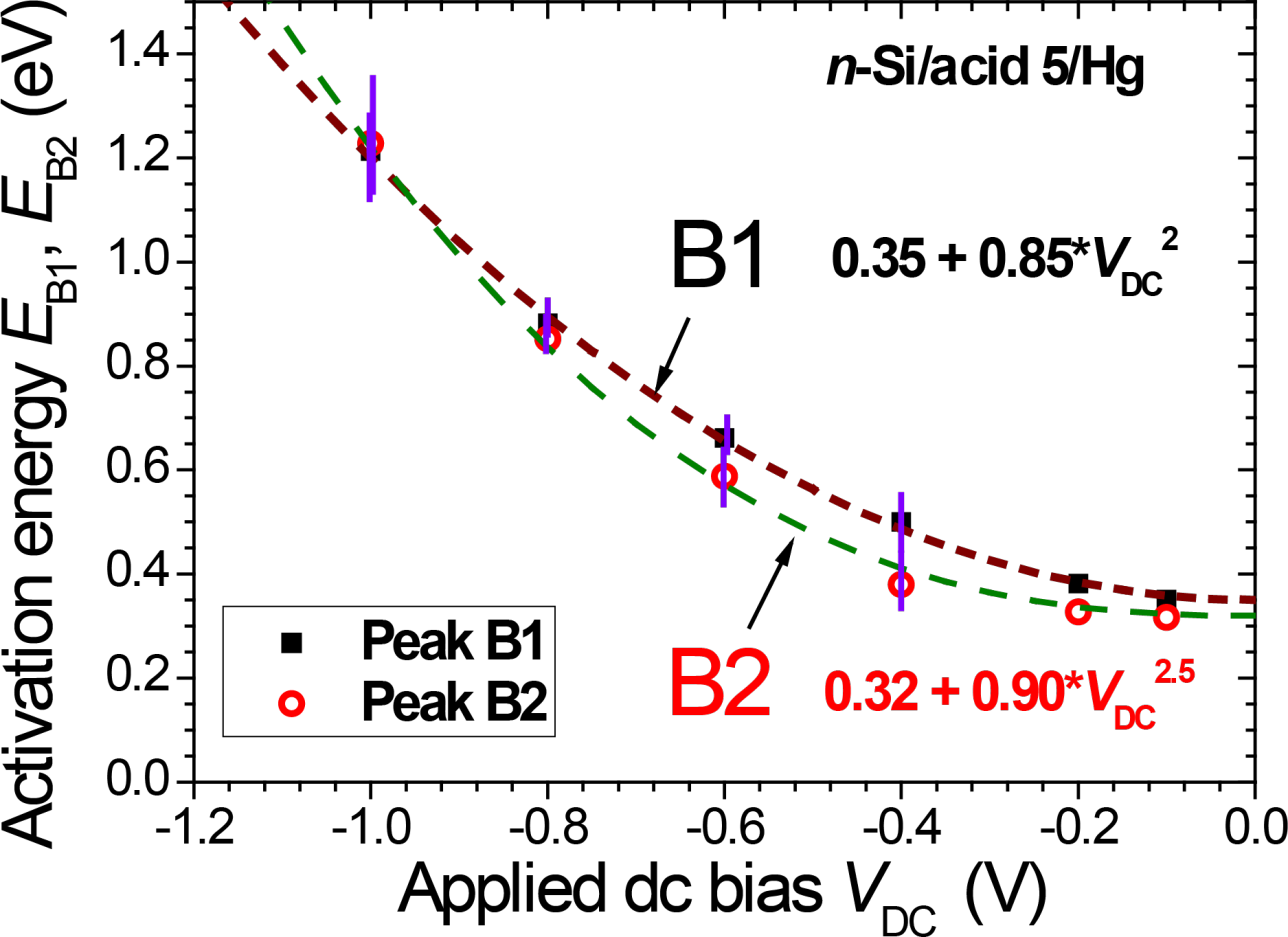
Bias dependence of dipolar relaxation activation energies, *E*_B1_ (squares) and *E*_B2_ (circles). The lines are power-law functions with forced exponent values: *E*_B1_ (eV) = 0.35 + 0.85 |*V*_DC_|^2^; *E*_B2_ (eV) = 0.32 + 0.90 |*V*_DC_|^2.5^.

Since the activation energies for relaxation peaks B1 and B2 both increase with the applied |*V*_DC_| values, the temperature fitting range becomes narrower and the resulting error bars increase. As a consequence, the analysis of relaxation data is limited to |*V*_DC_| ≤ 1 V. The resulting activation energies, *E*_B1_ and *E*_B2_ summarized in Figure 5, strongly depend on the applied dc bias, with a superlinear behavior. The bias dependence is slightly stronger for *E*_B2_ as compared to *E*_B1_. Both activation energies can be reasonably described by power law functions |*V*_DC_|*^q^*, with *q ≥* 2. If the exponents *q*_1_ = 2.0 and *q*_2_ = 2.5 are forced in this power-law dependence, one obtains *E*_B1_ (eV) = 0.35 + 0.85 |*V*_DC_|^2^ and *E*_B2_ (eV) = 0.32 + 0.90 |*V*_DC_|^2.5^ (Figure 5).

**Figure 6:**
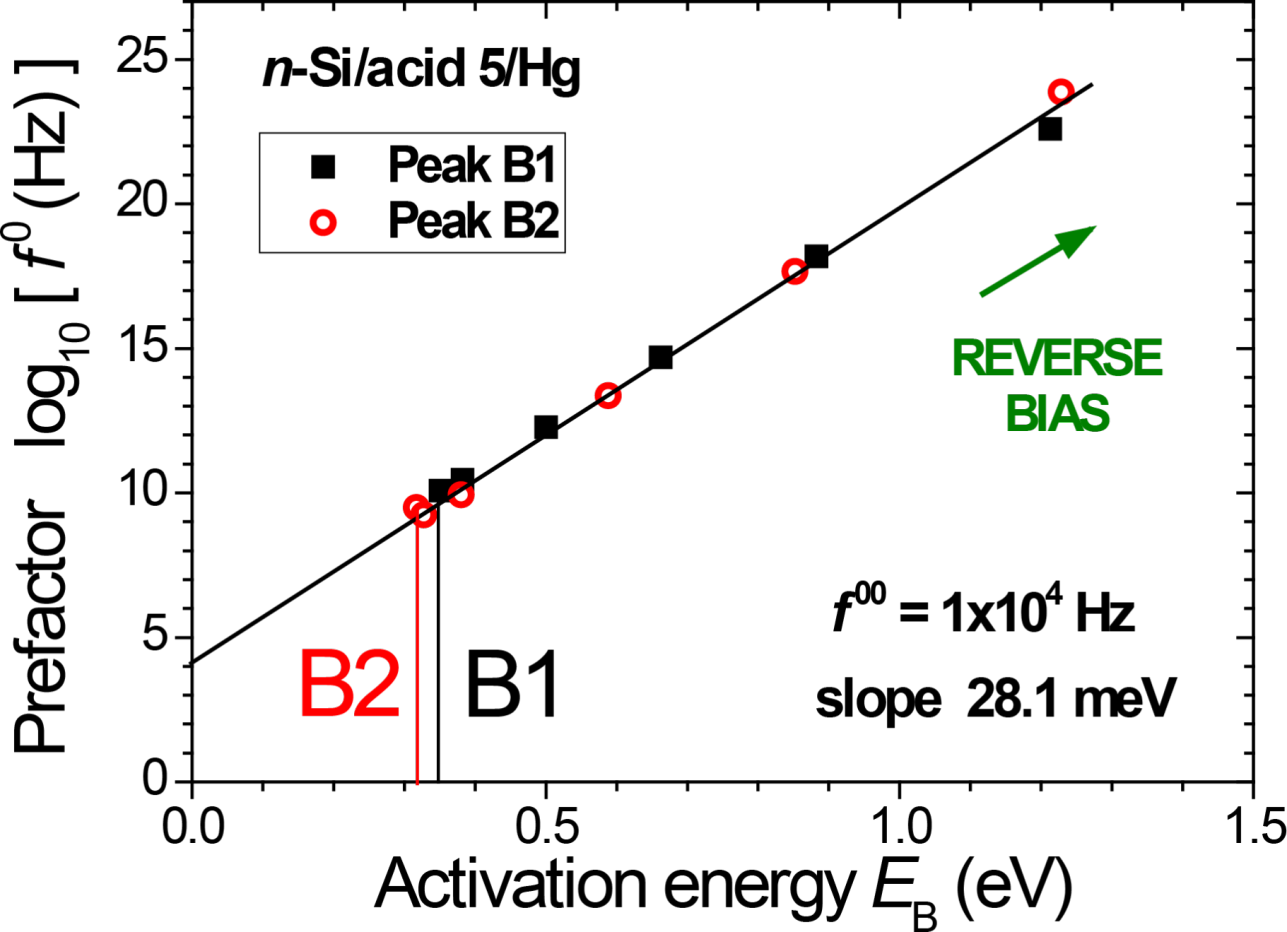
Linear correlation between activation energy and pre-exponential factor values derived from Figure 4, for peaks B1 (squares) and B2 (circles). The inverse slope is kT* = 28.1 meV (T* = 325 K).

A strong correlation between activation energy (in the range of 0.3–1.3 eV) and pre-exponential factor (range 10^10^ to 10^24^ Hz) is found in Figure 6, showing a linear dependence of log *f*_B_^0^(*V*_DC_) vs *E*_B_(*V*_DC_), up to very high values of the pre-exponential factor. A remarkable feature is that both relaxation mechanisms follow the same linear correlation, with inverse slope kT* = 28.1 meV (T* = 325 K). The similar values found for the inverse slope temperature T* (Figure 6) and the "focal point" temperature *T*_F_ given by Arrhenius plots (Figure 4) will be discussed below.

**Figure 7:**
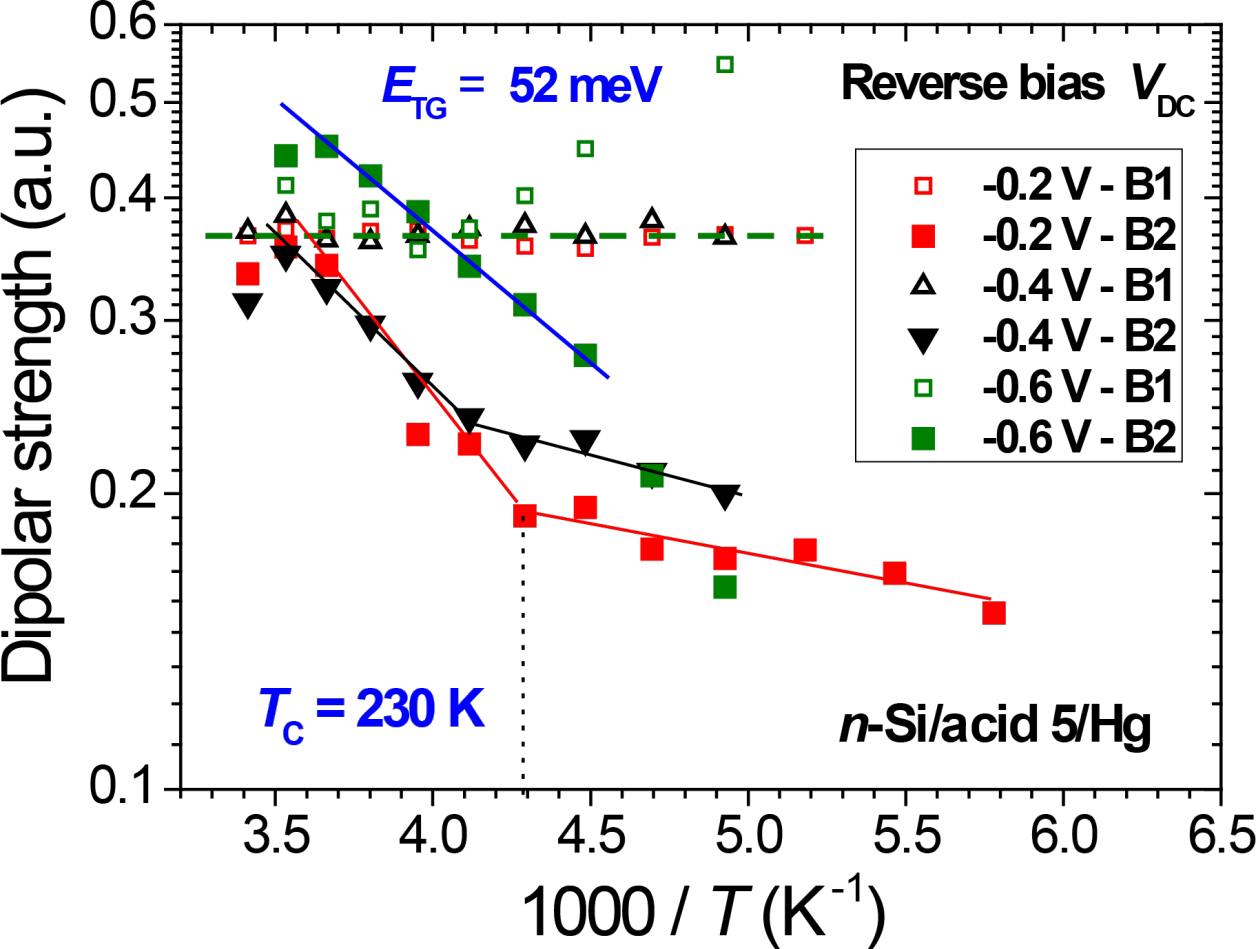
Temperature dependence of the dipolar relaxation strength Δε of peaks B1 and B2, measured at reverse bias values *V*_DC_ = −0.2, −0.4, and −0.6 V. At low applied bias, two different regimes appear, below and above 230 K.

**Dipolar relaxation strength:** Since the rather weak peak B2 overlaps with peaks B1 and A, respectively, at lower and higher frequencies, the fitted strength, Δε, of peak B2 is lower than its apparent value at the peak frequency, as shown in Figure 4. The dipolar relaxation strengths of mechanisms B1 and B2 show a different behavior as a function of the temperature, as illustrated in Figure 7 for several reverse bias values. For a given *V*_DC_ value, whereas the peak strength of B1 is basically constant, the peak strength of B2 increases (by a factor of about three) with increasing temperature in the range from 130 to 280 K; for smaller applied bias values, a larger slope is observed above 230 K.

## Discussion

The dynamics of the Si/acid 5 junction shows two different relaxation mechanisms with similar activation energies, both increasing with applied reverse dc bias. In this section, we attribute both relaxation mechanisms to dipolar units in the OML, namely acid end groups (B1) and gauche defects (B2), and we argue that the electrostatic pressure (induced by the dc voltage drop over the insulating monolayer) is responsible for both increasing motional constraints and increasing the number of gauche defects. In addition, both dipole relaxation mechanisms reveal a strong coupling of the dipole reorientation path with the molecular vibrational modes and manifest a collective dynamic response.

### Trans–gauche isomerization

Over the whole investigated temperature range, the strength of relaxation peak B1 is remarkably stable (Figure 7), as expected for a constant number of carboxylic acid dipoles. This indicates that the weak electrostatic pressure does not induce strong effects on their dipolar strength, which could arise, e.g., from a collapse of the organic monolayer.

In contrast, mechanism B2 attributed to gauche defects is enhanced at high temperatures and high dc bias. This temperature dependence supports previous modeling of C_18_ alkyl monolayers tethered to Si(111), showing an increase of gauche defects density by a factor of about five over the range 100–300 K [17]: (i) At low temperatures, little space is available for molecular reorientations and trans–gauche isomerization is restricted. (ii) With increasing temperature the number of chain gauche conformers rises and some disordering originates in the chain end regions and propagates towards the middle of the chain. The latter calculations are also consistent with vibrational and NMR spectroscopies [3,19,31,54].

In this admittance study, peak shapes do not show any evidence of ordering at low temperatures. Large values of the pre-peak slopes (*m*_B1_ ≈ *m*_B2_) are characteristic of long-range disorder for both mechanisms B1 and B2. In contrast, the post-peak slopes (*n*_B1_ < *n*_B2_) indicate that the chain ends (B1) are more disordered while they simultaneously experience larger motional constraints (smaller frequency, larger activation energy) than the gauche defects (B2).

Assuming a constant dipolar strength for all gauche defects, the dependence of the gauche defect response on temperature (range 220–280 K) provides a value of 52 ± 5 meV for the activation energy of Δε (B2) (Figure 7). This trans–gauche isomerization energy for tethered *n*-alkyl chains is larger than the value deduced from the density of vibrational states for short *n*-alkanes in the liquid phase (34 meV) [55–56] and comparable to that of perfluoro-*n*-alkanes (44 meV) [57].

However, an accurate quantification of the number of gauche defects would require an estimate of the dipole moments for all non-centrosymmetric methylene conformations, which will depend on details of the trans–gauche sequences. Vibrational spectroscopy and molecular dynamics studies of tethered alkyl monolayers have shown that the distribution of gauche defects at internal chain positions (kinks, gauche–gauche) and at chain ends (end-gauche) depends both on chain packing, chain length and temperature [58–59]. Interestingly, for the smaller applied bias, a weaker temperature dependence of Δε (B2) is observed below 230 K. Note that for longer *n*-alkyl tethered chains (C_12_ and above), a steady increase of the number of internal gauche defects was found from 100 to 300 K, while the density of end-gauche defects was very weakly dependent on the temperature [59]. The slope change observed near 230 K in Figure 7 may thus reveal the presence of end-gauche conformers at low temperatures, and an increase in internal gauche configurations (including kinks and double-gauche conformers) at higher temperatures. However, additional defects arising from disordered domain boundaries cannot be excluded.

### Multiexcitation entropy model of relaxation frequency

With increasing reverse bias, slowing down of both relaxation frequencies *f*_B1_ and *f*_B2_ due to motional constraints is related to a simultaneous increase of both activation energy (*E*_B_) and pre-exponential factor (*f*_B_^0^) values. Hence, a kind of "compensation" occurs, namely the frequency decrease is less dramatic than that which would arise from a change in activation energy only. More precisely, we have shown in Figure 6 that the relaxation mechanisms B1 and B2 follow the same linear correlation (with inverse slope kT*) between *E*_B_ (in the range of 0.3–1.3 eV) and log(*f*_B_^0^) (in the range from 10^10^ to 10^24^ Hz). The latter apparent prefactor values exceed typical phonon frequencies (10^13^ Hz) by more than ten decades.

This "compensation law" observed for solid-state phenomena with a large activation energy in many areas (physics, mechanics, chemistry, biology) has been rationalized by a multi-excitation entropy model, in the strong coupling limit, which incorporates the entropy of low energy excitations collected from a thermal bath [47]. In the YMC model, when the entity which is being excited and the excitation reservoir (the lattice and its vibrations) are so strongly coupled as to be indistinguishable, the excitation must be treated by using the transition state theory and the Eyring equation, with a free energy for the transition, Δ*G* = Δ*E* − *T*Δ*S* that includes the large entropy associated with this fluctuation. Hence, the resulting frequency expression *f*_B_ = *f*_B_^0^ exp(Δ*S*/*k*)·exp(−Δ*E*/*kT*) may explain very large apparent prefactor values, *f*_B_^0^ exp(Δ*S*/*k*); a linear relationship between Δ*S* and Δ*E* is thus a sufficient condition to obtain a linear compensation law (as found in Figure 6).

In the YMC model, when a large activation enthalpy barrier Δ*E* is obtained from a bath of elementary excitations, the number necessary to pass the barrier can be estimated from *n* ≈ Δ*E/h*ω*,* where *h*ω is the average energy of the bath excitations (typically tens of meV). To evaluate the number Ω of configurational paths, considered as equally probable, Yelon, Movaghar and Crandall consider an interaction volume [48] containing *N* excitations, where *N* is the number that must be available in order to provide the *n* excitations to be annihilated. The change in entropy


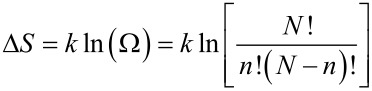


is approximated by


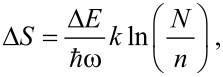


if *N* >> *n* (Stirling’s approximation). Since the larger the value of *N*, the more likely the process, the YMC model emphasizes that *N* may be treated as a coupling constant, between the external force and the microscopic motion at the molecular level. If the coupling with the thermal bath is not strongly dependent on Δ*E*, this relation predicts the existence of a “focal point” in the Arrhenius plot at the temperature


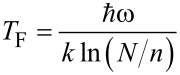


along with a linear relationship between activation energy and logarithm of the pre-exponential factor with slope *kT*_F_.

In the framework of the multiexcitation entropy model, the temperature T* = 325 K found in this work provides the energy (28 meV or 227 cm^−1^) of the collected excitations available from the thermal bath (divided by a number of the order of 1). Hence, as |*V*_DC_| increases, increasingly large number of vibrational excitations (in the range of 10–50) is required to overcome the activation barrier for collective molecular reorientation.

### Coupling with vibrational modes

Thermal bath excitations (227 ± 10 cm^−1^) revealed by dipolar relaxation dynamics can be compared with vibrational energies of *n*-alkanes, either in the liquid phase or in a two-dimensionally tethered phase.

For *n*-alkanes in the liquid phase, low vibration energies correspond to skeletal deformations, i.e., torsion or expansion/contraction [60]. Calculated spectra of the density of vibrational states for short *n*-alkanes, taking into account the distribution of conformational configurations resulting from the trans–gauche isomerization [55–56], show a C–C stretching band centered around 900 cm^−1^, obviously too high to account for our results, whereas torsion modes of CH_2_–CH_2_ units occur at too low energies (150 cm^−1^). In contrast, the CCC bending region centered near 220 cm^−1^ is related to the LAM-1 mode (single node longitudinal acoustic mode), which is also called “accordion” mode because all CCC angles change in phase. Disorder effects in the LAM modes were studied by Raman spectroscopy; the frequency in the solid ordered phase (all-trans configuration) is smaller (200 cm^−1^) than in the liquid phase (broad band near 240 cm^−1^) in which a disordered-LAM (or D-LAM) scattering is taken as the envelope of a statistical distribution of LAM-1 modes of short trans planar sequences [61].

The first LAM-*k* frequencies (*k* being the number of nodes) have been computed for *n*-alkane molecules of variable length tethered to a solid surface [62], showing very good agreement of the C_12_ alkane LAM-1 frequency (226 cm^−1^) with our results. Some molecular dynamics studies of tethered *n*-alkanes also reveal similar vibrational energies in the velocity autocorrelation function [63].

In summary, the dynamics of the Si/acid 5 junction reveal a cooperative coupling of thermal excitations to dipolar relaxation of the tethered *n*-alkane OML. The characteristic wavenumber of 227 cm^−1^ is related to the longitudinal acoustical mode (LAM-1) of the chain skeleton rather than to the higher energy CC stretching mode. Since the LAM frequency of the accordion mode is length dependent [62], this model predicts that the "focal point" temperature *T*_F_ should decrease with increasing *n*-alkane length.

### OML mechanics: compressive and shear forces

In this section, the molecular layer is considered as a continuous medium submitted to a compressive electrostatic pressure, proportional to *V*_I_^2^, where *V*_I_ is the potential drop across the insulating OML with thickness *d*_OML_ and dielectric permittivity ε_I_:

[2]
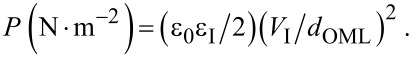


This electrostatic force leads to compressive and shear stress components, respectively, normal and parallel to the substrate.

Furthermore, each molecule is considered as a rigid elastic rod, tilted at some angle with respect to the normal to the surface and submitted to forced oscillations. The mechanical response to normal compression load has been tabulated for a number of geometries [49]. For a rod that is fixed on one side and free on the other side (fixed–free conditions), as occurs for linear tethered molecules, stress and strain depend on the spring constant *k* = (*P*/*E*·*I*)^1/2^ where *I* is the moment of inertia of the rod, *E* its Young modulus, and *P* the normal compression load.

In the context of a molecular layer submitted to compression, conformational changes of the molecules must be taken into account (Figure 1). A gauche defect (B2) such as a kink conformer is depicted as a laterally displaced bond with respect to the plane of the alkyl chain, while a tilted chain corresponds to a global angular displacement of the molecule backbone axis and hence of the end-group dipole (B1). Specific transverse shear moments result from these two situations [49]. In the case (B2) of a rod submitted to axial compression *P* plus externally created lateral displacement Δ_0_, the transverse shear is proportional to the product (*P*·*k*), i.e., *P*^3/2^, because the angular tilt θ_A_ ≈ Δ_0_·*k*. In the case (B1) of a rod submitted to axial compression plus externally created angular displacement θ_0_, the transverse shear is simply proportional to *P*, assuming that the angular tilt θ_A_ ≈ θ_0_ is independent of axial compression.

This simple nano-mechanical model for the fixed–free rod conditions thus predicts a stronger normal pressure dependence of the transverse shear for (kink) gauche defects as compared to end group dipoles. This corresponds qualitatively to the larger sensitivity to the applied bias found for *E*_B2_ as compared to *E*_B1_, shown in Figure 5. Although |*V*_I_| is expected to increase with |*V*_DC_|, the fact that *V*_I_ is not directly measured in our experiments precludes further conclusions.

## Conclusion

This fundamental admittance spectroscopy study provides new guidelines to identify the contribution of activated dipolar relaxation mechanisms and to discriminate the response of electrically active interface defects in organic monolayer/semiconductor assemblies.

Dynamic properties of tethered *n*-alkyl molecules were studied using a Hg/C_12_H_25_/n-type Si(111) junction with partial substitution of methyl end groups by polar acid moieties. Two temperature-activated relaxation mechanisms have been attributed to dipole end groups (B1) and gauche defects (B2). The temperature dependence of peak B2 dipolar strength provides a trans–gauche isomerization energy of 50 meV for tethered *n*-alkyl molecules. The enhanced B2 peak density, observed with increasing applied bias, is attributed to the formation of gauche defects as a means of reducing the stress due to electrostatic pressure across the nanometer-thick insulating layer. This effect is expected to be stronger with shorter alkyl chains (thinner OML in Equation 2) with possible implications on electron transport through molecular tunnel barriers. Further work is required to investigate its dependence on the OML packing density.

Arrhenius plots of relaxation frequencies show that activation energies and pre-exponential factors strongly increase with applied dc voltage, |*V*_DC_|. This "compensation law", which governs the relaxation frequencies for both dissipation mechanisms (Figure 6), reveals a strong coupling of end-group dipoles and gauche defects with the longitudinal acoustic mode (LAM-1, bending vibration) of the C_12_ alkyl chain. In the context of a multi-excitation entropy model, the isokinetic temperature *T*_F_, related to the energy of elementary excitations of the bath (here the *n*-alkyl accordion-like vibration), is expected to decrease with increasing *n*-alkyl chain length.

Since entropic contributions in the cooperative backbone mobility of tethered molecular layers also appear in the friction dissipation processes (coupling between external shear and internal molecular modes of relaxation) [24], a comparative study of tribological and electrostatic compression responses might be fruitful.

This collective dynamic behavior of alkyl chains tethered to Si is also consistent with the asymmetric relaxation peak shapes related to many-body interactions in complex relaxing systems. In this study, peak shape analysis indicates better short-range order (along with weaker motional constraints) for gauche defects as compared with end-group dipoles.

## Experimental

### Covalent immobilization of mixed alkyl/acid OML

Covalent OML grafting was performed on hydrogen-terminated Si(111):H surfaces using linear alkene molecules with a UV-assisted liquid phase process [40,64]. A low-doped n-type Si (phosphorus doped, 1–10 Ω·cm resistivity, Siltronix) was chosen to obtain rectifying junctions. After etching, the Si(111):H surface was used immediately for covalent binding of the mixed *n*-dodecyl/undecanoic acid-terminated monolayer (denoted as Si/acid 5), using the photochemical reaction at 300 nm for 3 h of Si(111):H with a mixture of undecylenic acid/1-dodecene (molar ratio 5/95).

### Admittance measurements

Admittance measurements were performed with a frequency response analyzer (Alpha-A High Resolution, Novocontrol Technologies). The sample holder was inserted in a two terminal active cell with the impedance converter mounted directly above the sample.

The junction was placed into a cryostat under dry nitrogen flow to avoid extensive water condensation and to minimize surface oxidation during electrical measurements [39–40]. An ohmic back contact was obtained by applying a silver paste electrode on the scratched Si rearside and a mercury top electrode (99.999% Fluka, contact area *S* = 5 × 10^−3^ cm^2^) was used to avoid electrical shorts through possible pinholes in the OML. A solid Hg electrode is obtained in the low temperature range (*T* < 233 K) but no discontinuity is observed in the junction properties at this particular temperature.

The ac modulation amplitude *V*_AC_ was set at 20 mV. The capacitance (4.5 pF) of the empty Teflon cell in parallel with the molecular junction was subtracted to obtain *C*_m_. At high frequencies, useful information on dipolar mechanisms is limited by the series resistance *R*_S_ (due to bulk Si and back contact resistance). Acquisition of dipolar relaxation data was performed in the reverse bias regime of the rectifying metal/OML/Si junction, with decreasing temperature steps and, at each temperature, the dc bias was scanned from the reverse regime (*V*_DC_ = −2 V) towards the forward regime (*V*_DC_ = +1 V).

The trans–gauche isomerization reactions are nearly reversible under our experimental conditions. To illustrate this point, the weak spectral changes observed upon return to room temperature (293 K) after the low temperature scan have been analyzed: peak B1 (respectively B2) is slightly shifted to higher frequency by a factor of 2.2 (respectively a factor of 2.8); while the peak strength ratio Δε(B2)/Δε(B1) has increased by about 10% (at −0.6 V).

## Acknowdedgements

Bruno Fabre (SCR, University of Rennes 1) is acknowledged for molecular grafting experiments. The author is also grateful to A.B. Fadjie-Djomkam and S. Ababou-Girard (IPR, University of Rennes 1) for their participation in the admittance measurements.
